# Toxicity of Plant Secondary Metabolites Modulating Detoxification Genes Expression for Natural Red Palm Weevil Pesticide Development

**DOI:** 10.3390/molecules22010169

**Published:** 2017-01-20

**Authors:** Ahmed Mohammed AlJabr, Abid Hussain, Muhammad Rizwan-ul-Haq, Hassan Al-Ayedh

**Affiliations:** 1 Laboratory of Bio-Control and Molecular Biology, Department of Arid Land Agriculture, College of Agricultural and Food Sciences, King Faisal University, Hofuf 31982, Al-Ahsa, Saudi Arabia; aljabr@kfu.edu.sa (A.M.A.); mianrizwan15@gmail.com (M.R.-H.); 2 Life Science and Environment Research Institute, King Abdulaziz City for Science and Technology, P.O. Box 6086, Riyadh 11442, Saudi Arabia; alayedh@kacst.edu.sa

**Keywords:** *cytochrome P450*, *esterases*, feeding indices, *glutathione S-transferase*, *Rhynchophorus ferrugineus*, toxicity

## Abstract

This study aimed to explore the larvicidal and growth-inhibiting activities, and underlying detoxification mechanism of red palm weevil against phenylpropanoids, an important class of plant secondary metabolites. Toxicity of α-asarone, eugenol, isoeugenol, methyl eugenol, methyl isoeugenol, coumarin, coumarin 6, coniferyl aldehyde, diniconazole, ethyl cinnamate, and rosmarinic acid was evaluated by incorporation into the artificial diet. All of the phenylpropanoids exhibited dose- and time-dependent insecticidal activity. Among all the tested phenylpropanoids, coumarin exhibited the highest toxicity by revealing the least LD_50_ value (0.672 g/L). In addition, the most toxic compound (coumarin) observed in the current study, deteriorated the growth resulting tremendous reduction (78.39%) in efficacy of conversion of digested food (ECD), and (ECI) efficacy of conversion of ingested food (70.04%) of tenth-instar red palm weevil larvae. The energy-deficient red palm weevil larvae through their intrinsic abilities showed enhanced response to their digestibility resulting 27.78% increase in approximate digestibility (AD) compared to control larvae. The detoxification response of *Rhynchophorus ferrugineus* larvae determined by the quantitative expression of *cytochrome P450*, *esterases*, and *glutathione S-transferase* revealed enhanced expression among moderately toxic and ineffective compounds. These genes especially *cytochrome P450* and *GST* detoxify the target compounds by enhancing their solubility that leads rapid excretion and degradation resulting low toxicity towards red palm weevil larvae. On the other hand, the most toxic (coumarin) silenced the genes involved in the red palm weevil detoxification mechanism. Based on the toxicity, growth retarding, and masking detoxification activities, coumarin could be a useful future natural red palm weevil-controlling agent.

## 1. Introduction

Red palm weevil, *Rhynchophorus ferrugineus* (Olivier) (Coleoptera, Curculionidae), is a severe pest of a wide genera of palms with a worldwide distribution [1,2]. Weevils destroy the vascular system of the palm trunk, which eventually lead towards the collapse and death of the infested palm [1]. The cryptic life cycle of weevils, which occurs inside the palm truck, presents serious management challenges [2]. Previously, the red palm weevil control strategy mainly depended on the use of synthetic insecticides. The intensive use of insecticides against the invaded populations of red palm weevil impart detrimental effects on non-target animals, environmental pollution, and insecticide residues in the food [3]. Recent reports on the continued low efficacy of insecticides due to the development of insecticide resistance in the most destructive pest of palm, *R. ferrugineus* is challenging for farmers, researchers, and policy-makers. These facts signify the importance on the development of alternative control approaches.

Different environmental friendly strategies including entomopathogenic fungi [4,5,6], nematodes [7], bacteria [8,9], development of pheromone traps [10,11,12,13], sterile insect technique [14,15,16,17], and plant based insecticides [18,19,20,21] remained the subject of previous research investigations. Among them, plant-based products especially plant secondary metabolites are the most promising alternatives to environmental deteriorating insecticides.

The metabolic pathways of plants generate tens of thousands of secondary products. These plant secondary metabolites have shown numerous benefits in different industries [22]. Currently, the untapped reservoir of plant secondary metabolites are of particular interest in insecticide development [23]. These natural compounds gaining importance in pest management due to various deleterious effects including anti-feedant, toxic, repellent, fumigant, attractant, molting disruption, respiratory inhibition, pheromone-based behavioral adaptations, oviposition deterrence, and fecundity reduction against target pest populations [23,24,25,26,27]. In the past, this broad-spectrum approach was ignored in red palm weevil control. Little research data of preliminary stages are available on the use of plant-based raw products including essential oils [18,28], plant extracts [20,29,30], plant metabolites, such as rotenone and limonine [31], against red palm weevils.

Phenylpropanoid plant secondary metabolites are synthesized by phenylalanine within the plants. These compounds (phenylpropanoids) have been isolated from throughout the plant kingdom. The insecticidal potential of different phenylpropanoids have been tested against different pest species in a number of investigations [32,33,34,35]. A literature survey has shown that there is no report on the use of phenylpropanoids against red palm weevils, thus, we decided for the first time, to investigate the insecticidal potential of phenylpropanoids. Our study aimed to (1) screen the toxicity of selected phenylpropanoid plant secondary metabolites including α-asarone, eugenol, isoeugenol, methyl eugenol, methyl isoeugenol, coumarin, coumarin 6, coniferyl aldehyde, diniconazole, ethyl cinnamate, and rosmarinic acid against red palm weevils; (2) determine the influence of phenylpropanoids on the feeding performance of *R. ferrugineus* larvae; and (3) explore the host detoxification mechanism against the toxicity of phenylpropanoids to pave the way for the isolation and development of eco-friendly compounds against red palm weevils.

## 2. Results

### 2.1. Dose Mortality Response

**Figure 1:**
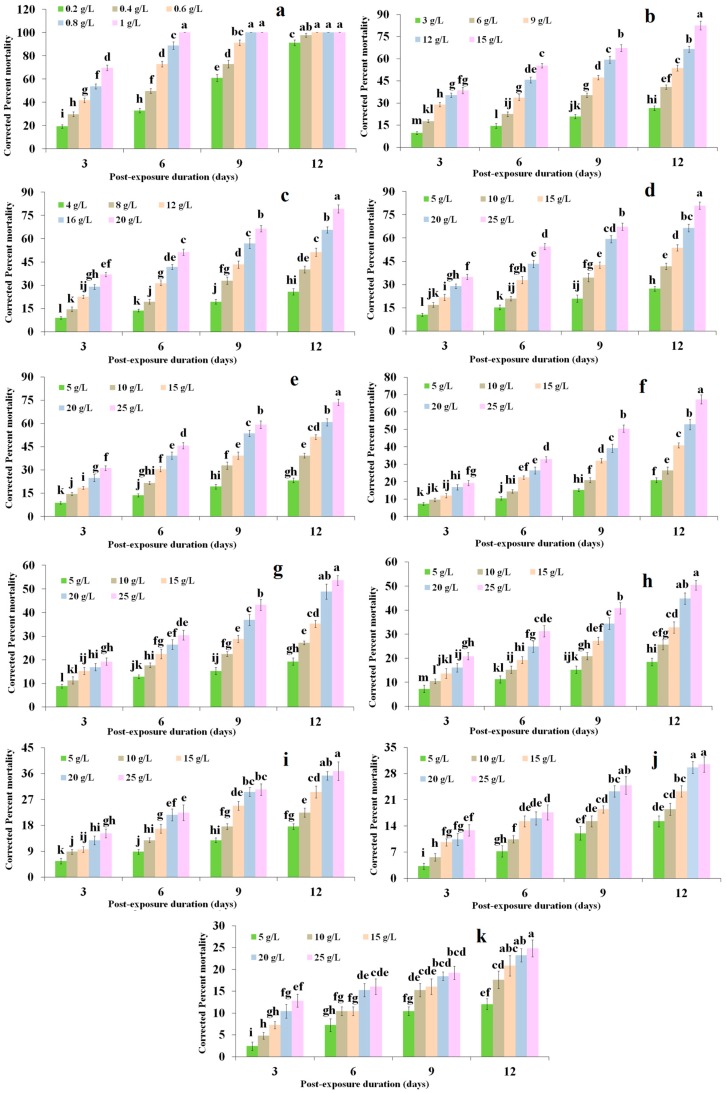
Percent corrected cumulative dose-mortality response of *R. ferrugineus* larvae against (**a**) coumarin; (**b**) ethyl cinnamate; (**c**) diniconazole; (**d**) isoeugenol; (**e**) coumarin 6; (**f**) asarone; (**g**) eugenol; (**h**) coniferyl aldehyde; (**i**) rosmarinic acid; (**j**) methyl isoeugenol; and (**k**) methyl eugenol. Bars (means ± SE) followed by different letters are significantly different. (Fisher’s LSD test, α = 0.05).

Toxicity of selected phenylpropanoids toward red palm weevil larvae varied significantly. However, only coumarin caused 100% mortality with the least LD_50_ value after three days (0.672 g/L); and found to be the most toxic phenylpropanoid in the current study (Figure 1; Table 1). Comparatively, coumarin at all tested doses (*F* = 893.67; df = 4, 64; *p* < 0.0001), time intervals (*F* = 189.63; df = 3, 64; *p* < 0.0001), and their interaction (*F* = 95.28; df = 12, 64; *p* < 0.0001) imparted significantly higher mortality (Figure 1). In our toxicity analysis, rosmarinic acid, methyl isoeugenol, and methyl eugenol showed very little promise in red pam weevil control because they failed to impart 50% mortality even at a dose of 25 g/L during the course of whole experimentation (Table 1). On the other hand, seven phenylpropanoids, including ethyl cinnamate, diniconazole, isoeugenol, coumarin 6, α-asarone, eugenol, and coniferyl aldehyde imparted > 50% mortality and their LD_50_ values calculated after 12 days ranged between 6 and 28 g/L (Table 1). However, the interaction of dose mortality response at different time intervals for ethyl cinnamate (*F* = 10.30; df = 12, 64; *p* < 0.0001), diniconazole (*F* = 6.42; df = 12, 64; *p* < 0.0001), isoeugenol (*F* = 9.65; df = 12, 64; *p* < 0.0001), coumarin 6 (*F* = 13.37; df = 12, 64; *p* < 0.0001), α-asarone (*F* = 4.64; df = 12, 64; *p* < 0.0001), eugenol (*F* = 3.22; df = 12, 64; *p* < 0.0001), and coniferyl aldehyde (*F* = 1.14; df = 12, 64; *p* < 0.0001) were significantly different (Figure 1). Overall, these seven phenylpropanoids due to high LD_50_ values and slow mode of action found to be less toxic to red palm weevil larvae.

### 2.2. Growth Inhibition Activities of Red Palm Weevil Larvae

The incorporation of each tested phenylpropanoid at a dose of 0.672 g/L into the artificial diet to calculate nutritional indices including ECI, ECD, and AD after 72 h of feeding have shown tremendous variations in their response. Red palm weevil larvae fed on diets incorporated with coumarin showed the highest reduction in ECD (78.39%), and remained significantly different to all other diets incorporated with other phenylpropanoids (*F* = 146; df = 11, 48; *p* < 0.0001). However, the lowest reduction (<15%) was observed from the diets incorporated with methyl eugenol, methyl isoeugenol, rosmarinic acid, coniferyl aldehyde, eugenol, and α-asarone (Table 2).

The most toxic compound (coumarin) screened in the current study also tremendously reduced (70.04%) the ECI of *R. ferrugineus* larvae. The remaining treatments failed to impart tremendous reduction, resulting in <12% reduction compared to the control treatment diet. However, significant differences in the ECI index among the tested compounds were observed (*F* = 170; df = 11, 48; *p* < 0.0001). Furthermore, the ECI index of methyl eugenol and methyl isoeugenol remained significantly at the same level compared to the control treatment diets (Table 2). Overall, inhibition in growth indices (ECI and ECD) directly proportional to the toxicity of phenylpropanoids. The highly toxic compound, coumarin, screened in the current study, imparted the highest inhibition in the ECI and ECD indexes. On the other hand, the least toxic compounds failed to inhibit growth indices of *R. ferrugineus* larvae. On the contrary, an inversely proportional relationship was observed between AD and toxicity of the isolates compared to the ECI and ECD indices (Table 2). The most toxic compound, coumarin, significantly enhanced (27.78%) the AD of *R. ferrugineus* larvae compared to the control treatment larvae. The artificial diets incorporated with all other tested phenylpropanoids failed to tremendously enhance AD. and resulted in <11% compared to control treatment larvae. However, AD significantly increased upon exposure to different treatments (*F* = 208; df = 11, 48; *p* < 0.0001).

### 2.3. Quantitative Reverse Transcription Polymerase Chain Reaction Analyses of Red Palm Weevil Detoxification Genes

**Figure 2:**
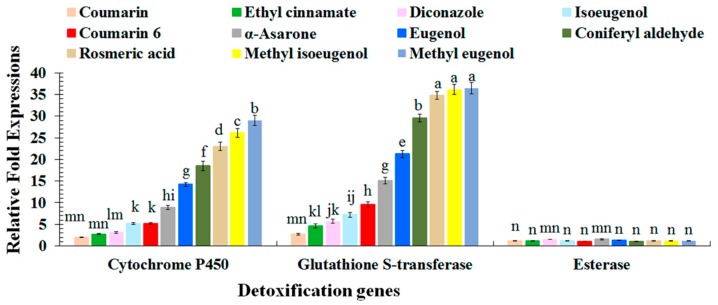
Expression pattern of detoxification genes of red palm weevil larval midguts in response to different phenylpropanoids by qRT-PCR. Bars (means ± SE) followed by different letters are significantly different (Fisher’s LSD test, α = 0.05).

Toxicity of tested plant phenylpropanoids induced different levels of detoxification (*cytochrome P450*, *glutathione S-transferase*, and *esterase*) genes (Figure 2). The expression of detoxification genes (*F* = 2269.48; df = 2, 132; *p* < 0.0001), upon feeding on different treatments (*F* = 500.51; df = 10, 132; *p* < 0.0001) and their interaction (*F* = 136.94; df = 20, 132; *p* < 0.0001), showed significant differences. Larvae fed on diet incorporated with methyl eugenol, methyl isoeugenol, and rosmarinic acid tremendously enhanced the expression of tested detoxification genes especially *GST* and *cytochrome P450* (Figure 2). Among all the detoxification genes, *GST* showed the highest expression. All of the treatments, except coumarin, greatly induced the expression of *GST* and *cytochrome P450* (Figure 2). However, *esterase* failed to induce expression and remained significantly at lower level compared to other tested detoxification genes.

## 3. Discussion

Red palm weevil control is facing a threat because of the development of insecticide resistance in *Rhynchophorus ferruginous* against conventional synthetic insecticides, warranting the development of newer plant-based eco-friendly insecticides [3]. Our screening program for discovering potent compounds was designed by evaluating toxicity, feeding performance and host detoxification against selected phenylpropanoids. We have reported for the first time the toxicity and growth retarding activities of coumarin that disturbed the genes that regulate detoxification mechanism.

**Figure 3:**
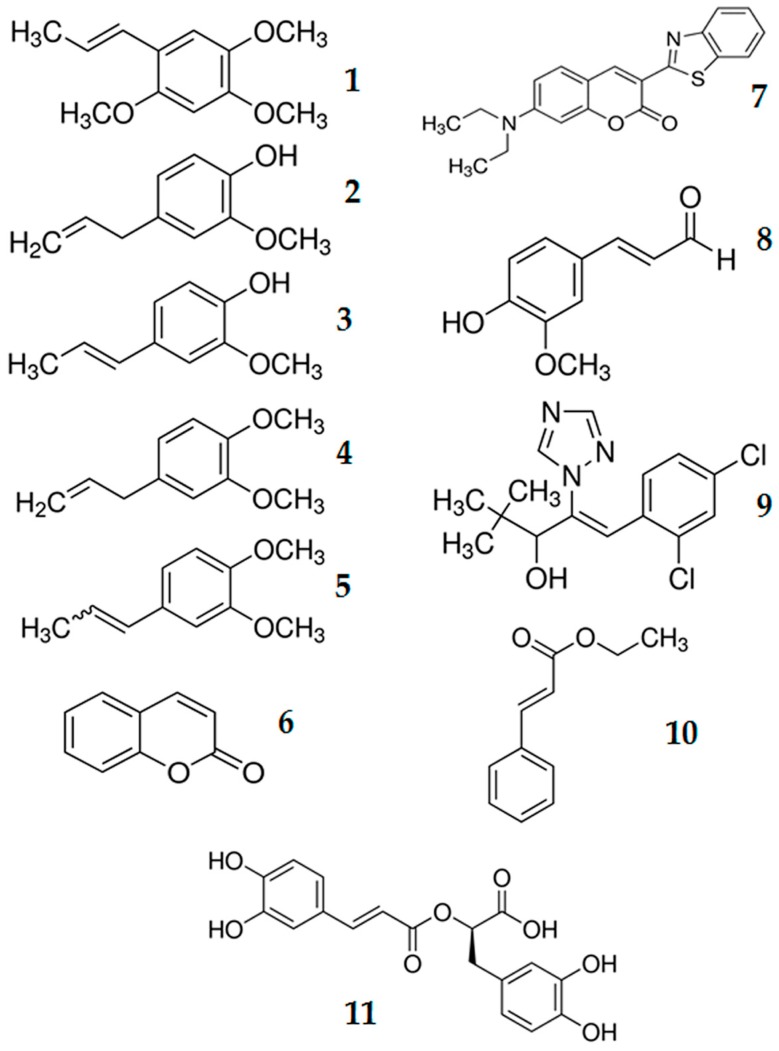
Chemical structures of α-asarone **1**, eugenol **2**, isoeugenol **3**, methyl eugenol **4**, methyl isoeugenol **5**, coumarin **6**, coumarin 6 **7**, coniferyl aldehyde **8**, diniconazole **9**, ethyl cinnamate **10**, and rosmarinic acid **11**.

Dietary laboratory experimentation of selected phenylpropanoids (Figure 3) against red palm weevil larvae exhibited various biological effects from low to high degrees. Among all the tested compounds, artificial diet incorporated with coumarin, showed acute toxicity, imparting the least LD_50_ (0.672 g/L) against red palm weevil larvae. The efficacy of coumarin is equivalent to the efficacy observed by Moreira et al. [34], who examined the effect of coumarin on coleopterons infesting stored products. They found coumarin as the best treatment against *Oryzaephilus surinamensis* (L), and *Rhyzopertha dominica* (F) at all the studied time intervals. The LD_50_ results reported by Moreira et al. [34], coincides with our findings against red palm weevil larvae. The LD_50_ of *R. dominica* reported by Moriera after 6 h was 39.71 mg/g (39,710 ppm), while after 12 h it was 20.82 mg/g (20,820 ppm) that was 47.57% decrease in LD_50_ value compared to 6 h LD_50_ value. Furthermore, LD_50_ value after 24 h was 11.82 mg/g (11,820 ppm) that was 70.23% decrease in LD_50_ value compared to 6 h LD_50_ value. The difference in LD_50_ values between our results against red palm weevil with *R. dominica* and *O. surinamensis* mainly because of the time to calculate LD_50_ values as reflected in the Table 1. However, the toxicity calculated through LD_50_ values of other phenylpropanoids ranged from moderate (ethyl cinnamate, diniconazole, isoeugenol, coumarin 6, α-asarone, eugenol and coniferyl aldehyde) to ineffective (rosmarinic acid, methyl isoeugenol, and methyl eugenol) against red palm weevil larvae. Toxicity results of rosmarinic acid, methyl isoeugenol, and methyl eugenol at the highest concentration (25 g/L) during the course of whole experimentation without attaining 50% mortality showed that these compounds have little promise to be used as bio-pesticide against *R. ferruginous* larvae.

Coumarin imparted significant reductions in the feeding performance of *R. ferrugineus* larvae, when it incorporated into the diet at a dose of 0.672 g/L. Tenth-instar *R. ferruginous* larvae fed on a coumarin-containing diet showed the least efficacy of conversion of ingested (ECI), and digested (ECD) diet, which meant that less food was available for growth because most of the energy was metabolized for host defense. Similar nutritional indices patterns of ECD and ECI in red palm weevil larvae were reported, when larvae were dipped in conidial suspensions of entomopathogenic fungi [4,5], or larvae fed on diets incorporated with synthetic pesticides [3]. Such tremendous reductions in ECI (70.04%), and ECD (78.39%) by coumarin compared to control larvae observed in the current study revealed that most of the energy from ingested food is being used to perform physiological activities to combat the toxin (coumarin), which means that less food is being utilized for larval growth and development. In contrast, coumarin 6, α-asarone, eugenol and coniferyl aldehyde, rosmarinic acid, methyl isoeugenol, and methyl eugenol failed to impart significant reductions in ECI and resulting less than 10% reduction in ECI compared to control suggesting low toxicity potential of these tested compounds against tenth-instar red palm weevil larvae. In addition, such minor reductions in ECD compared to control were observed when larvae were fed on diet incorporated with rosmarinic acid (10.36%), methyl isoeugenol (5.26%), and methyl eugenol (2.42%). Previous research reported the contact and fumigation action of methyleugenol, (*E*)-methyl isoeugenol, and α-asarone against *Liposcelis bostrychophila* Badonnel [33]. Their findings reported much lower LD_50_ (125.73 μg/cm^2^) of α-asarone by contact toxicity bioassays compared to our LD_50_ (17.670 g/L) results suggesting its little potential in controlling red palm weevil larvae.

Bioactivity of the most potent compound (coumarin) evaluated in the current study greatly enhanced (27.78%) the approximate digestibility (AD) compared to the control treatment *R. ferrugineus* tenth-instar larvae. The enhanced response of AD values among coumarin-fed *R. ferrugineus* larvae unveil the fact of the use of some extra energy for host defense. In order to fulfill this energy shortfall, treated larvae through their intrinsic abilities enhanced their approximate digestibility of the limited foodstuff. Previous laboratory investigations also reported similar enhanced AD response among *R. ferrugineus* larvae to pesticides [3]; and fungal conidia [4,5]; *Ocinara varians* larvae to fungal suspensions [36].

The physiological responses of *R. ferrugineus* larvae promote the expression of genes involved in the detoxification of toxic phenylpropanoids by enhancing metabolic mechanisms of target pest. Hussain et al. [3] reported the enhanced activity of *GST* among resistant populations of red palm weevil fed on artificial diet incorporated with cypermethrin. Among all the detoxification genes evaluated in the current study, *GST* gene was highly expressed indicating that *GST* rapidly detoxify the compounds especially α-asarone, eugenol, coniferyl aldehyde, rosmarinic acid, methyl isoeugenol, and methyl eugenol resulting more than 10% *GST* expression. Similar enhanced expression was also reported in the current investigations in the case of *cytochrome P450*. However, their expression remained significantly lower compared to *GST* expression (Figure 2). In the past, the expression of *GST, cytochrome P450*, and *esterase* against toxicants in other pest species is well documented [37,38,39]. The high expression of *GST* and *cytochrome P450* directly related with the detoxification of host against the tested compounds. These genes detoxify the target compounds by enhancing their solubility that leads rapid excretion and degradation [40]. The lethality of phenylpropanoids are greatly reduced by the enhanced expression of detoxification genes especially *GST* and *cytochrome P450*. The most toxic compound (coumarin) target *GST* and *cytochrome P450* genes resulting silencing/masking their expression that ultimately lead to the lowest LD_50_ value (0.672 g/L). In contrast, *esterase* studied in the current investigations did not greatly stimulate the tested compounds and their expression remained very little. The low expression does not necessarily mean that these genes are not involved in host defense against toxicant. Furthermore, it cannot be ruled out that low expression genes might involve in defense to other toxicants not tested.

## 4. Materials and Methods

### 4.1. Insects

The red palm weevils (*R. ferruginous*) were obtained from the laboratory cultures previously collected from the date palm infested fields in October-December 2014. Adults were shifted to pineapples in cages (57.5 cm × 29 cm × 58 cm) for mating and oviposition at 30 ± 1 °C, 75% ± 5% relative humidity (RH), with a 16-h light photoperiod. Neonates were fed on pineapples. However, second instar *R. ferrugineus* larvae were shifted individually to an artificial diet in 400 mL perforated plastic cups at 30 ± 1 °C, 75% ± 5% relative humidity (RH) with a 16-h light photoperiod [4].

### 4.2. Phenylpropanoid Plant Secondary Metabolites

Eleven phenylpropanoids, including α-asarone, eugenol, isoeugenol, methyl eugenol, methyl isoeugenol, coumarin, coumarin 6, coniferyl aldehyde, diniconazole, ethyl cinnamate, and rosmarinic acid, based on their toxicity to other pest species as shown in Figure 3 were purchased from Sigma Aldrich, London, UK.

### 4.3. Toxicity Bioassays

In the laboratory, range-finding studies were performed to decide the appropriate doses of phenylpropanoids against red palm weevil larvae. The pure compounds were dissolved in acetone and diluted in distilled water. Five doses of each tested compound were incorporated into the artificial diet to determine the toxicity of red palm weevil larvae. Control treatment diet was prepared using similar volume of acetone and distilled water. Twenty-five tenth-instar red palm weevil larvae were used for each treatment. Each larva was singly-fed on a treated artificial diet in aerated plastic bowls at 30 ± 1 °C, 75% ± 5% relative humidity (RH) with a 16-h light photoperiod. Five replicates from different red palm weevil generations for each treatment and control were prepared to compile toxicity bioassays. Dose mortality data were recorded daily. Toxicity bioassays were repeated over time on different red palm weevil generations. The observed mortality data of red palm weevil larvae were corrected for control mortality by commonly used Abbott’s formula [41]. All replicate results were subjected to Probit analysis to determine LD_50_ values using POLO software (LeOra Software LLC, Berkeley, CA, USA) [42]. Angularly transformed corrected cumulative mortality data at different time intervals were analyzed by repeated measures ANOVA with Fisher’s LSD test [43].

### 4.4. Nutritiopnal Indices of Red Palm Weevils

Feeding performance was calculated with tenth-instar red palm weevil larvae as these larger larvae and their frass could easily be handled and accurately weighed. Diets were prepared separately using LD_50_ value for each compound based on toxicological laboratory bioassays. In this experiment, twenty-five red palm weevil tenth-instar larvae per replicate were separately provided with artificial diet incorporated with the phenylpropanoids in aerated plastic bowls at 30 ± 1 °C, 75% ± 5% relative humidity (RH) with a 16-h light photoperiod. Among all of the treatments, coumarin was found to be the most potent compound. For nutritional indices, artificial diets were supplemented with either α-asarone, eugenol, isoeugenol, methyl eugenol, methyl isoeugenol, coumarin, coumarin 6, coniferyl aldehyde, diniconazole, ethyl cinnamate, and rosmarinic acid at the dose of 0.672 g/L based on the LD_50_ value of the most potent treatment (coumarin) as determined in laboratory toxicology bioassays against same instar (tenth) red palm weevil larvae. Five replicates from different red palm weevil generations for each treatment and control were prepared for feeding performance bioassays. After three days of experimentation, weight gained by each larva was accurately measured by analytical weight balance. In addition, food consumed and frass produced by each larva was determined by analytical weight balance. These measurements were used to determine nutritional indices of tenth-instar *R. ferrugineus* larvae. Nutritional indices including, efficacy of conversion of digested food (ECD = weight gained by the larva/(food ingested by the larva-dry weight of frass excreted by larvae)), efficacy of conversion of ingested food (ECI = 100 × dry weight gained by the larva/dry weight of food consumed by larva), and approximate digestibility (AD = (food ingested − frass weight)/food ingested × 100) were calculated as described in previous experimentation [4,5]. Significant differences between growth nutritional indices generated from larvae fed on diets incorporated with different phenylpropanoids were determined using one-way ANOVA and Fisher’s LSD test using SAS [44].

### 4.5. Host Detoxification Mechanism

The expression profiles of detoxification genes were validated by real-time quantitative PCR from the midgut portion of the alimentary canal of tenth-instar red palm weevil larvae, as these larger larvae have enough midgut to extract total RNA from single larva. In this exploration, red palm weevil (tenth-instar) larvae were fed in separate aerated plastic bowls on each treatment diet prepared by incorporating each tested phenylpropanoid compound separately at a dose of 0.672 g/L based on the LD_50_ value of the most potent treatment (coumarin) as determined in laboratory toxicology bioassays against same instar (tenth) red palm weevil larvae. The experimental units were kept at 30 ± 1 °C, 75% ± 5% relative humidity (RH) with a 16-h light photoperiod. Seventy-two hours post-feeding, tenth-instar larvae were dissected in saline for subsequent grinding of mid-gut portion in liquid nitrogen. A commercially available RNeasy® Universal Mini Kit (Qiagen, Hilden, Germany) was used to extract total RNA from five larvae and each regarded as one replicate. The extracted total RNA of each treatment was reverse-transcribed using PrimeScript First Strand cDNA Kit (TaKaRa Clontech, Paris, France). This template (first strand cDNA) of each treatment along with specific primers (Table 3) were used to quantify target gene expression in CFX96 Touch^TM^ (Bio-Rad, London, UK) using SYBR^®^ Premix Ex Taq™ II kit (TaKaRa Clontech, Paris, France). The numerical values obtained from each experimental unit were compared with those of the control by relative fold expression obtained by transforming the obtained results into absolute values using 2^−ΔΔCt^ [45]. In case of control, the relative expression of each gene was set to 1. *beta-Actin* (GenBank accession #KM438516) due to its low expression variations was chosen as housekeeping gene. The mid-gut samples extracted from red palm weevil larvae fed on different diets were analyzed by one-way ANOVA and the significant differences between means were determined by applying Fisher’s LSD test [44].

## 5. Conclusions

In conclusion, feeding red palm weevil larvae on coumarin tremendously reduced their survival, adversely affected their growth and development, and inhibited the expression of detoxification genes, including *GST* and *cytochrome P450*. These characteristics suggest coumarin as a biologically-active valuable source for future eco-friendly commercial development of coumarin against the infestations of red palm weevils.

## Figures and Tables

**Table 1:** Dose mortality response of red palm weevil larvae against different phenylpropanoids.

Treatments	LD_50_ (g/L)	95% CL	χ^2^	Slope ± SE
Coumarin ^1^	0.672	0.580–0.778	4.51	1.91 ± 0.25
Ethyl cinnamate ^2^	6.974	6.113–7.955	5.60	2.07 ± 0.25
Diniconazole ^2^	9.756	8.531–11.158	4.42	1.99 ± 0.25
Isoeugenol ^2^	11.560	10.080–13.270	4.50	1.98 ± 0.25
Coumarin 6 ^2^	13.330	11.580–15.350	1.57	1.87 ± 0.25
α-Asarone ^2^	17.670	15.040–20.750	5.94	1.80 ± 0.25
Eugenol ^2^	23.260	18.020–30.030	1.81	1.43 ± 0.25
Coniferyl aldehyde ^2^	27.010	19.740–36.970	1.58	1.34 ± 0.25
Rosmarinic acid ^3^	n/a	n/a	n/a	n/a
Methyl isoeugenol ^3^	n/a	n/a	n/a	n/a
Methyl eugenol ^3^	n/a	n/a	n/a	n/a

^1^ LD_50_ values were calculated after three days of feeding. ^2^ LD_50_ values were calculated after 12 days of feeding. ^3^ could not attain LD_50_ values because the treatments could not cause 50% mortality within the course of whole experimentation.

**Table 2:** Nutritional indices of red palm weevil larvae against different phenylpropanoids.

Treatments	AD	ECI	ECD
Coumarin	74.01 ± 0.35 ^a^	05.75 ± 0.53 ^f^	07.76 ± 0.71 ^h^
Ethyl cinnamate	60.02 ± 0.15 ^b^	15.01 ± 0.17 ^e^	25.01 ± 0.30 ^g^
Diniconazole	59.02 ± 0.44 ^bc^	16.01 ± 0.11 ^d^	27.13 ± 0.20 ^f^
Isoeugenol	58.06 ± 0.50 ^cd^	16.88 ± 0.24 ^c^	29.09 ± 0.62 ^e^
Coumarin 6	57.71 ± 0.32 ^de^	17.34 ± 0.21 ^bc^	30.05 ± 0.53 ^de^
α-Asarone	57.17 ± 0.49 ^de^	17.52 ± 0.19 ^bc^	30.66 ± 0.60 ^cde^
Eugenol	56.72 ± 0.40 ^ef^	17.69 ± 0.30 ^b^	31.20 ± 0.73 ^cd^
Coniferyl aldehyde	55.92 ± 0.25 ^fg^	17.71 ± 0.27 ^b^	31.68 ± 0.62 ^cd^
Rosmarinic acid	55.66 ± 0.33 ^g^	17.91 ± 0.10 ^b^	32.19 ± 0.31 ^c^
Methyl isoeugenol	55.34 ± 0.45 ^gh^	18.81 ± 0.40 ^a^	34.02 ± 0.99 ^b^
Methyl eugenol	54.33 ± 0.36 ^hi^	19.03 ± 0.30 ^a^	35.04 ± 0.74 ^ab^
Control	53.45 ± 0.28 ^i^	19.19 ± 0.21 ^a^	35.91 ± 0.58 ^a^

Means ± SE values having the same letter(s) within the column are not significantly different (Fisher’s LSD test, α = 0.05).

**Table 3:** Target genes and primer sequences used for quantitative PCR expression analysis of detoxification genes from red palm weevil larvae.

Target Gene	Accession No	Amplicon Size	Primer 5′–3′ (Forward and Reverse)
Detoxification			
*Cytochrome P450*	KT748789	118 bp	TGGAGAAACACCCGCAAGAA CGGCGATTTTGCCTACCAAG
*Glutathione S-transferase*	KR902496	92 bp	ATAGCCAACCACCACTGTCG CGTTCCTCTTGCCGCTAGTT
*Esterase*	KT748822	70 bp	ACCTACAAGAATCCGACGCC ACTCCGAAACTTTGGGCCAT
Housekeeping			
*beta-Actin*	KM438516	129 bp	AAAGGTTCCGTTGCCCTGAA TGGCGTACAAGTCCTTCCTG
